# Genome-wide analysis of the transcriptional response to porcine reproductive and respiratory syndrome virus infection at the maternal/fetal interface and in the fetus

**DOI:** 10.1186/s12864-016-2720-4

**Published:** 2016-05-20

**Authors:** Jamie M. Wilkinson, Hua Bao, Andrea Ladinig, Linjun Hong, Paul Stothard, Joan K. Lunney, Graham S. Plastow, John C. S. Harding

**Affiliations:** Department of Agricultural, Food, and Nutritional Science, University of Alberta, Edmonton, AB Canada; Department for Farm Animals and Veterinary Public Health, University Clinic for Swine, University of Veterinary Medicine, Vienna, Austria; Department of Large Animal Clinical Sciences, Western College of Veterinary Medicine, University of Saskatchewan, Saskatoon, SK Canada; Animal Parasitic Diseases Laboratory, Beltsville Agricultural Research Center, Agricultural Research Service, U.S. Department of Agriculture, Beltsville, MD USA; Key Lab of Agricultural Animal Genetics, Breeding and Reproduction of Ministry of Education, College of Animal Science and Technology, Huazhong Agricultural University, Wuhan, China

**Keywords:** RNA-sequencing, Transcriptome, Pig, Fetus, PRRSV, Gene

## Abstract

**Background:**

Porcine Reproductive and Respiratory Syndrome Virus (PRRSV) infection of pregnant pigs can result in congenital infection and ultimately fetal death. Little is known about immune responses to infection at the maternal-fetal interface and in the fetus itself, or the molecular events behind virus transmission and disease progression in the fetus. To investigate these processes, RNA-sequencing of two sites, uterine endothelium with adherent placental tissue and fetal thymus, was performed 21 days post-challenge on four groups of fetuses selected from a large PRRSV challenge experiment of pregnant gilts: control (CON), uninfected (UNINF), infected (INF), and meconium-stained (MEC) (n = 12/group). Transcriptional analyses consisted of multiple contrasts between groups using two approaches: differential gene expression analysis and weighted gene co-expression network analysis (WGCNA). Biological functions, pathways, and regulators enriched for differentially expressed genes or module members were identified through functional annotation analyses. Expression data were validated by reverse transcription quantitative polymerase chain reaction (RTqPCR) carried out for 16 genes of interest.

**Results:**

The immune response to infection in endometrium was mainly adaptive in nature, with the most upregulated genes functioning in either humoral or cell-mediated immunity. In contrast, the expression profile of infected fetal thymus revealed a predominantly innate immune response to infection, featuring the upregulation of genes regulated by type I interferon and pro-inflammatory cytokines. Fetal infection was associated with an increase in viral load coupled with a reduction in T cell signaling in the endometrium that could be due to PRRSV-controlled apoptosis of uninfected bystander cells. There was also evidence for a reduction in TWIST1 activity, a transcription factor involved in placental implantation and maturation, which could facilitate virus transmission or fetal pathology through dysregulation of placental function. Finally, results suggested that events within the fetus could also drive fetal pathology. Thymus samples of meconium-stained fetuses exhibited an increase in the expression of pro-inflammatory cytokine and granulocyte genes previously implicated in swine infectious disease pathology.

**Conclusions:**

This study identified major differences in the response to PRRSV infection in the uterine endometrium and fetus at the gene expression level, and provides insight into the molecular basis of virus transmission and disease progression.

**Electronic supplementary material:**

The online version of this article (doi:10.1186/s12864-016-2720-4) contains supplementary material, which is available to authorized users.

## Background

Porcine Reproductive and Respiratory Syndrome Virus (PRRSV) causes respiratory disease and slow growth in growing pigs, and reproductive failure in pregnant sows through abortions, stillbirths, and the farrowing of weak-born piglets that have a high mortality rate. Nearly 25 years after its discovery [1, 2], it remains one of the largest infectious disease concerns of pig farmers worldwide. PRRSV is a single-stranded RNA virus that evolves at a rapid rate through the accumulation of random mutations and intragenic recombination events. There is therefore considerable genetic and antigenic heterogeneity among PRRSV strains [3]. The virus replicates in cells of the monocyte/macrophage lineage, from where it can suppress innate antiviral immune mechanisms and delay the onset of an effective adaptive immune response using a variety of immune evasion strategies. The course of infection typically consists of two phases: an acute, viraemic phase that lasts several weeks, and a chronic infection of lymphoid tissues that can persist for many months before it is cleared [4]. The combination of this virus’ extensive genetic diversity and its immunosuppressive characteristics has made it difficult to design vaccines that are effective in the event of heterologous challenge. Consequently, there is substantial interest in characterizing the host response to infection at the transcriptomic and genomic level, to help design a new generation of more immunogenic vaccines, and to identify potential natural resistance mechanisms in swine populations.

A considerable number of transcriptomic experiments of PRRSV infection have been conducted. These studies adopted either an *in vitro* infection model of macrophages focusing on the first hours of infection following virus entry into the cell [5–7], or *in vivo* challenge models that usually considered a longer infection time-course [8–13]. They encompass a variety of designs from standard time-course experiments to the comparison of viral strains of divergent virulence or pigs of differential susceptibility to infection, but all these experiments investigated the respiratory form of the disease in growing pigs. The reproductive form of PRRS has yet to be investigated with this technology.

Experimental inoculation of sows in late gestation results in transplacental infection and gross fetal pathology that is consistent with that observed in the field during PRRS outbreaks, whereas inoculation earlier in gestation does not typically result in fetal infection [14, 15]. The reason for this and the mechanism of transplacental viral transmission are not well understood. Messenger RNAs for TNF-α and IFN-γ are transcribed in fetal tissues upon PRRSV infection, indicating that the fetus is capable of mounting an immune response [16], but detailed information on the nature of the immune response and the cause of any fetal pathology is lacking.

We have recently conducted the largest PRRSV challenge experiment in pregnant pigs yet undertaken [17]. Pregnant gilts were inoculated at 85 days of gestation and euthanized 21 days later to collect samples from 111 PRRSV-inoculated and 19 mock-inoculated gilts and their fetuses for a variety of assays, including transcriptomic analysis. The objectives of this study were to use a transcriptomics approach to investigate immune responses to infection in the reproductive tract and the processes of transplacental infection and fetal pathology. To this end, the present study utilized tissue samples taken from the endometrium (including adherent placental layers) and fetal thymus of selected fetuses. The selection of these two tissues was strategic. The maternal/fetal interface is the site at which virus transmission occurs, whereas thymus is one of the proposed sites of primary viral replication in the fetus [16]. Gene expression in these tissues was then examined through a series of pair-wise comparisons across four groups of fetuses: control (CON - uninfected fetuses from mock-inoculated gilts), uninfected (UNINF – uninfected fetuses from PRRSV-inoculated gilts), infected (INF – infected fetuses with no external signs of pathology from PRRSV-inoculated gilts), and meconium-stained (MEC – meconium-stained, infected fetuses from PRRSV-inoculated gilts). MEC fetuses were found almost exclusively in litters from PRRSV-inoculated gilts and the MEC fetuses from those litters had very high viral loads. The MEC classification was presumed therefore to represent a stage in PRRSV-induced fetal pathology. The pairwise comparisons were UNINF v CON, INF v UNINF, and MEC v INF. The purpose of the UNINF v CON contrast was to elucidate responses to PRRSV infection of endometrium in the absence of fetal infection. The purpose of the INF v UNINF contrast was to identify responses in fetal thymus associated with fetal infection, whereas in endometrium it was to identify responses that may be associated with transplacental infection. The purpose of the MEC v INF contrast was to explore the progression of disease in both endometrium and fetal thymus. Specifically, our hypotheses were that the nature of the immune response to infection in endometrium and fetus is broadly similar; and that fetal pathology is largely a consequence of damage to the maternal/fetal interface.

## Methods

### Experimental infection of pregnant gilts with PRRSV

The animal experiment described herein was approved by the University of Saskatchewan’s Animal Research Ethics Board, and adhered to the Canadian Council of Animal Care guidelines for humane animal use (protocol #20110102). A more detailed version of the ethics statement and experimental protocol has previously been described [17]. Briefly, 133 Landrace gilts were obtained from a high-health nucleus herd that was free of PRRSV according to clinical signs and serologic testing. Groups of gilts were selected over 12 bi-weekly replicates, estrus-synchronized, and bred to Yorkshire boars. Pregnant gilts were housed in stalls until gestation day 80 ± 1, and then transferred to a biosafety level-2 facility. After a period of acclimation, 114 gilts were inoculated on gestation day 85 ± 1 (Experiment day 0) with 1x10^5^ TCID_50_ type 2 PRRSV isolate NVSL 97–7895. Nineteen control gilts were mock inoculated in a similar manner with Minimal Essential Medium. Gilts were humanely euthanized on Experiment day 21.

### Categorization and sampling of fetuses

The gravid uterus of each gilt was removed intact, placed in a trough, and orientated from left oviduct to right oviduct. The uterine horns were opened and fetuses numbered sequentially according to their position within the horn. The preservation status of each fetus was recorded *in situ* as: viable (live at the time of gilt necropsy, normal pink appearance), meconium-stained (live at time of gilt necropsy, stained with meconium), decomposed (dead prior to gilt necropsy, with primarily white skin), autolysed (dead prior to gilt necropsy, with over 50 % brown-colored skin), or mummified (dead, <20 cm in length and considered dead prior to PRRSV-inoculation). Mummified fetuses were counted, but excluded from sampling and further analyses. Each fetus was removed from the uterus together with its umbilical cord and a portion of allantochorion and uterus adjacent to the umbilical stump. Multiple samples of select tissues were then obtained from each individual: endometrium (including adherent placental layers) adjacent to the umbilical attachment site, fetal thymus, and blood from the axillary artery (from viable and meconium-stained fetuses only) from which serum was subsequently separated. Samples for transcriptomics were snap-frozen in liquid nitrogen and immediately stored at -80 °C. Samples for quantification of virus were immediately stored at -80 °C, and samples for histopathology were stored in 10 % formalin. To prevent cross-contamination of nucleic acid, necropsy instruments and surfaces were disinfected in ‘Synergize’ (Pro-AG products Ltd, Winnipeg, Canada) for at least 10 min between animals (gilts and fetuses).

### Quantification of PRRSV RNA and histopathology

PRRSV RNA concentrations were measured in endometrium, fetal thymus, and fetal serum using a strain-specific, in-house NVSL 97–7895 specific quantitative reverse transcription polymerase chain reaction (qRT-PCR) as described in detail elsewhere [17]. Results were reported as log_10_ target RNA concentration per mg or μL. A semi-quantitative scoring system was used to categorize the viral RNA concentration in each tissue sample: 0 = no detectable virus, 1 = positive, but non-quantifiable amounts of viral RNA, 2 = positive and less than mean concentration of similar quantifiable tissue, and 3 = positive and greater than mean concentration of similar quantifiable tissue. For histopathology, samples of endometrium and fetal thymus were collected in 10 % formalin and processed for hematoxylin and eosin staining from all live fetuses. Tissues were examined for the presence or absence of characteristic lesions associated with *in utero* PRRS infection. A more detailed description is provided elsewhere [18].

### Fetus selection and isolation of RNA for transcriptomic experiments

A subset of fetuses (4 groups of 12 individuals) was selected for transcriptomics experiments. The four groups were: ‘control’ (CON: fetuses from mock-infected gilts), ‘uninfected’ (UNINF: PRRSV-negative fetuses from infected gilts), ‘infected’ (INF: infected fetuses from infected gilts), and ‘meconium-stained’ (MEC: meconium-stained fetuses from infected gilts). CON fetuses were selected from randomly chosen gilts (one fetus per gilt) and were all PRRSV RNA negative (score 0). The remaining three groups were blocked for gilt. UNINF fetuses were viable and had a PRRSV score of 0 in both thymus and serum, INF fetuses were viable and had a PRRSV score ≥ 2 in thymus, and MEC fetuses were meconium-stained and had a PRRSV score of ≥ 2 in thymus. Where possible, UNINF, INF, and MEC fetuses were selected from the same uterine horn. Differences in log_10_ mean viral RNA concentration between groups and tissues were determined using Student’s T test.

Total RNA was isolated from 10–20 mg of endometrium and thymus of each fetus using an ‘All Prep RNA/DNA isolation Mini kit’ (Qiagen, Hilden, Germany) according to the manufacturer’s instructions. RNA was quantified by spectrophotometry using a Nanodrop ND 2000 (Thermo Fisher Scientific, Waltham, USA). RNA quality was assessed by digital electrophoresis using a ‘2200 Tapestation’ (Agilent Technologies, Santa Clara, USA). The mean RNA Integrity Number (RIN) was 9.0 for thymus (range 8.0–9.8) and 8.3 for endometrium samples (range 7.5–8.8).

### Library construction, RNA-sequencing, and generation of expression data

Ninety-six cDNA libraries were constructed for RNA-sequencing (RNA-seq) from thymus and endometrium samples of individual fetuses. Libraries were prepared from 1 μg of total RNA using the TruSeq RNA sample preparation kit v2 (Illumina, San Diego, USA) according to the manufacturer’s instructions. DNA libraries were sequenced at the Genome Quebec Innovation Centre (McGill University, Montreal, Canada). Library insert size distribution was assessed on a LabChip GX instrument (Perkin Elmer, Waltham, USA) using a high sensitivity chip. Library concentration was determined using a KAPA Library Quantification Kit (KAPA Biosystems) on a Lightcycler 480 II instrument (Roche Life Science, Indianapolis, USA). Cluster formation was performed on the cBot instrument (Illumina) with eight libraries per lane (7 pM per library) using the TruSeq PE Cluster Kit v3-cBot-HS (Illumina). Paired-end 100 bp sequencing was performed on a HiSeq 2000 (Illumina) using the TruSeq SBS kit v3-HS (Illumina). Real-time analysis and base calling were performed using the Hi-seq Control Software, version 2.2.38 (Illumina) to generate sequence reads with associated base quality scores. RNA-seq reads, flagged as low quality by CASAVA 1.8 (Illumina), or with an average PHRED read quality score below 15, or in which ≥5 of the last 10 bases had a quality score below 2, were removed. Reads were aligned to the pig reference genome sequence assembly (Sscrofa10.2) using TopHat 1.4.0 with default parameters [19]. The gene annotation information used for Sscrofa10.2 was from Ensembl 71. The number of reads uniquely mapped to each gene was determined using Ht-seq count (v0.5.3.p3) [20].

### Analysis of differential gene expression and gene co-expression networks

Differentially expressed genes (DEG) were identified using the Bioconductor package ‘edgeR’ [21]. Raw expression counts were converted to counts per million (CPM). Genes with very low expression levels were filtered out of the dataset by setting an expression threshold of CPM > 1 in at least 12 samples. Compositional differences between libraries due to difference in library size were normalized using the ‘Trimmed mean of M values’ (TMM) method. A negative binomial model was fitted to the data and gene and tag-wise dispersions were estimated using the quantile-adjusted conditional maximum likelihood (qCML) method. Differential expression was determined using an Exact test for the following three contrasts for both tissues: UNINF v CON, INF v UNINF, and MEC v INF. The purpose of the INF v UNINF contrast was to identify responses in fetal thymus associated with fetal infection, whereas in endometrium it was to identify responses that may be associated with transplacental infection. The purpose of the MEC v INF contrast was to explore the progression of disease in both endometrium and fetal thymus. Genes were classified as differentially expressed if they had an absolute fold change >1.5 and a False Discovery Rate (FDR) <0.05. Venn diagrams for the DEG lists for the 3 contrasts in each tissue were constructed in Venny [22].

Clustering of samples was performed by multidimensional scaling in two dimensions using the plotMDS function in the bioconductor package ‘limma’ [23]. Samples were clustered according to the Euclidian distance between samples pairs for the 500 genes with the largest SD between samples.

Gene co-expression network analysis was performed using the software package ‘WGCNA’ [24] for each of the three contrasts in the two tissues described above. Normalized CPM expression data generated by ‘edgeR’ were transformed using the function log_2_ (x + 1) to stabilize variance prior to use in WGCNA. To construct the network, a matrix of co-expression similarity for all genes across all samples in each contrast was made. Next, a weighted network adjacency matrix was obtained by raising the co-expression similarity to a power β whose value was determined by application of the approximate scale-free topology criterion [25]. Average linkage hierarchical clustering using the topological overlap measure of network interconnectedness identified clusters of highly interconnected gene modules. Network construction and module detection were performed using the function ‘blockwiseModules’, with the following parameter values: a maximum block size of 15000 (sufficiently large to analyze all genes in a single block), a minimum module size of 30, a medium sensitivity to cluster splitting of 2, and a cut height for module merging of 0.25. The value of β ranged from 6–10. The gene expression profile of a module can be summarized by the module eigengene, defined as the first principal component of the expression matrix. Correlations between the module eigengene and the two categorical traits in each contrast were determined. The threshold for association significance was set at an absolute correlation of >0.3 and a *P* value of <0.1.

### Gene function enrichment analysis

The DEGs (from edgeR) and significant modules (from WGCNA) from all contrasts were analyzed using Ingenuity Pathway Analysis (IPA) (Ingenuity Systems, Redwood City, USA) software to identify biological functions and pathways that were enriched in those sets of genes. Ensembl IDs from the Sscrofa 10.2 genome build annotation were used to identify the orthologous human gene for input into IPA. All novel genes, pseudogenes, and transcripts without a human orthologue were removed at this stage. Gene IDs and either log_2_ fold changes (for DEGs) or correlations (WGCNA module genes) were then imported into IPA, and mapped to their corresponding objects in the Ingenuity Knowledge Database (IKB). Gene sets were analyzed using the Biological Function, Canonical Pathways, and Upstream Regulator analysis tools. Analyses used a right-tailed Fisher’s Exact Test to identify functions/pathways/regulators that were enriched in the gene set compared to the reference set (all genes in the human genome) for that function/pathway/regulator. For the biological function and canonical pathway analyses, an FDR ≤ 0.05 cutoff was used for statistical significance; for upstream regulator analysis the significance threshold was set at *P* < 0.01. Each analysis also used a Z score metric to indicate the degree of consistency between the actual versus expected direction of expression change among genes annotated with that function/pathway/regulator based on data in IKB. An unbiased Z score of ≥2 was used to define a function/pathway/regulator as ‘Activated’, while a score of ≤ -2 defined an ‘Inhibited’ function/pathway/regulator. A breakdown of DEGs by Gene ontology (GO) slim molecular function and biological process terms across all contrasts was produced using the PANTHER function classification tool [26].

### Validation of expression data by Reverse Transcription Quantitative Polymerase Chain Reaction (RT-qPCR)

The expression profiles of selected genes of interest from the RNA-seq analyses were validated using an RT-qPCR approach. Primer and probe sequences were either obtained from pre-existing, validated assays in the DGIL Porcine Translational Research Database (www.ars.usda.gov/Services/Docs.htm?docid=6065) or newly designed from the porcine genome (Sscrofa v10.2). All assays were pre-tested on thymus or endometrium RNA pools (including CON, UNINF, INF, and MEC samples) to ensure that they met criteria for reaction efficiency, specificity, and sensitivity, as verified by the ABI Taqman 7500 software v2.3 (Applied Biosystems, Foster City, USA). After quality control, RT-qPCR assays were carried out for 12 genes in endometrium and 11 in thymus (16 genes total; 7 genes were assayed on both tissues). *RPL32* was chosen as a reference gene on the basis of its consistent expression between CON, UNINF, INF, and MEC groups observed by both RNA-seq and RT-qPCR. A list of primer and probe sequences used for these assays, synthesized by Integrated DNA Technologies (Coralville, USA), is provided in Additional file 1.

Reverse transcription reactions were carried on all samples used for RNA-seq (96 in total) using Superscript II Reverse Transcriptase (Invitrogen, Carlsbad, USA). For each sample, 1 μg of total RNA was used for each 20 μL reverse transcription reaction. All cDNA samples were preserved at -20 °C. qPCR reactions were performed in 96 well plates using the 7500 Real-Time PCR System (Applied Biosystems, Foster City, USA). The PCR reaction volume was 25 μl consisting of 25 ng cDNA, 3.6 μM primers, 0.5 μM probe, 2x Brilliant II qPCR Master Mix with low ROX (Applied Biosystems), and nuclease-free water. PCR conditions were as follows: 1 cycle of 10 min at 95 °C, followed by 40 cycles of 15 sec at 95 °C and 60 sec at 60 °C.

All RT-qPCR data for expression analysis were analyzed using the ΔΔCT method [27]. The statistical significance of differential expression was evaluated by Student’s T test. A significance level of *P* < 0.05 was considered significant.

## Results and Discussion

### Assessment of viral load and pathology across fetal groups and tissues

**Fig. 1:**
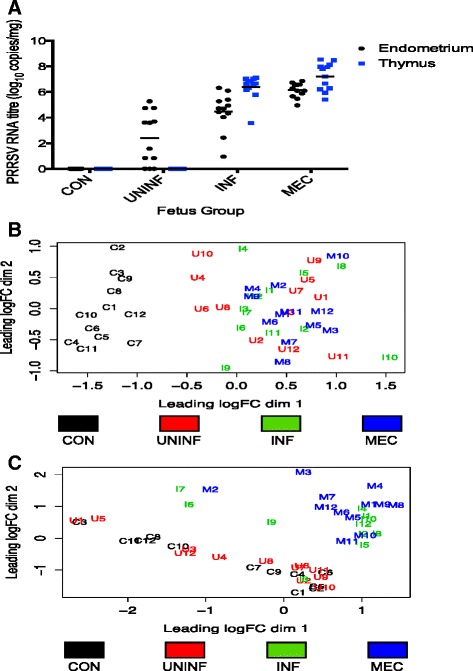
Comparison of individual endometrium and thymus samples. **a** PRRSV RNA concentration (target copies per mg tissue) in endometrium and fetal thymus for each of the 4 groups of fetuses used for transcriptomic analysis. **b** and **c** Cluster analysis (multidimensional scaling plots) on the basis of genome-wide gene expression in endometrium (**b**) and fetal thymus (**c**) for individual fetuses from each of the 4 groups used for transcriptomic analysis

Viral RNA concentrations varied considerably between groups and tissues (Fig. 1a). No virus was detected in any of the CON samples, as expected. The UNINF fetuses contained no viral RNA in thymus as this was a requirement of their classification, but the endometrial samples associated with this group had a wide range of viral RNA concentration (0-5.27 log_10_ RNA copies; mean = 2.41). All endometrium and thymus samples from INF and MEC pigs were PRRSV positive. Mean levels of viral RNA in endometrium were significantly different between each of the four groups (all *P* < 0.01), with levels in MEC > INF > UNINF > CON. In thymus, viral load was greater in INF and MEC than UNINF (both *P* < 0.001), and tended to be greater in MEC than INF fetuses (*P* = 0.056). For INF and MEC groups, viral RNA concentration was higher in thymus than endometrium (all *P* < 0.01). In terms of pathology, all fetuses from PRRSV-inoculated gilts (UNINF, INF, and MEC) had PRRSV-associated lesions in endometrium. In contrast, none of the thymus samples from inoculated gilts had PRRSV-associated lesions. No lesions were found in any of the CON samples of either tissue [18].

Six layers of uterine and placental tissues separate fetal and maternal blood supplies in the porcine reproductive tract. Infection of maternal tissue is therefore a necessary pre-requisite to infection of the fetal compartment. The successive increases in endometrial PRRSV RNA concentrations moving between the UNINF, INF, and MEC groups suggests that viral load in this tissue is an important contributor to fetal infection. Indeed, using the complete set of fetuses from all inoculated gilts we have previously shown that PRRSV RNA concentration in endometrium and thymus of live fetuses are highly correlated, and that endometrial PRRSV RNA concentration is the factor most predictive of viral load in fetal thymus and ultimately the probability of fetal death [28]. Whereas viral load in both tissues is evidently associated with disease progression in the fetus, this is not the case for presence of PRRSV-associated lesions. These were absent from all these fetal thymus samples, but present in all the endometrial samples, and these findings were consistent with those of the larger dataset of fetuses from all PRRSV-inoculated gilts [18]. Taken alone, these data support the hypothesis that fetal death is a consequence of PRRSV-associated pathology to the endometrium and placental attachment site [16, 29], however evidence from the transcriptional profile of meconium-stained fetuses suggests that this may not be so clear cut, and is discussed in a later section.

### Sample cluster analysis

Cluster analysis of thymus and endometrium samples based on their gene expression profiles was carried out using a multidimensional scaling method. The results clearly demonstrated that viral infection was the predominant factor affecting gene expression. The endometrium samples separated into two distinct clusters with the CON fetuses in one and the UNINF, INF, and MEC fetuses in the other (Fig. 1b). The three UNINF endometrium samples that did not contain any detectable PRRSV RNA (U4-U6) clustered with the UNINF, INF, and MEC rather than CON groups. The thymus samples also separated into two major clusters, but on the basis of the infection status of the fetus: with the CON and UNINF fetuses in one cluster and the INF and MEC fetuses in a second (Fig. 1c). However, one INF fetus (I3), the sample with the lowest viral load in that group (0.95 log_10_ RNA copies), clustered with the CON and UNINF groups.

### Overview of differential gene expression

**Fig. 2:**
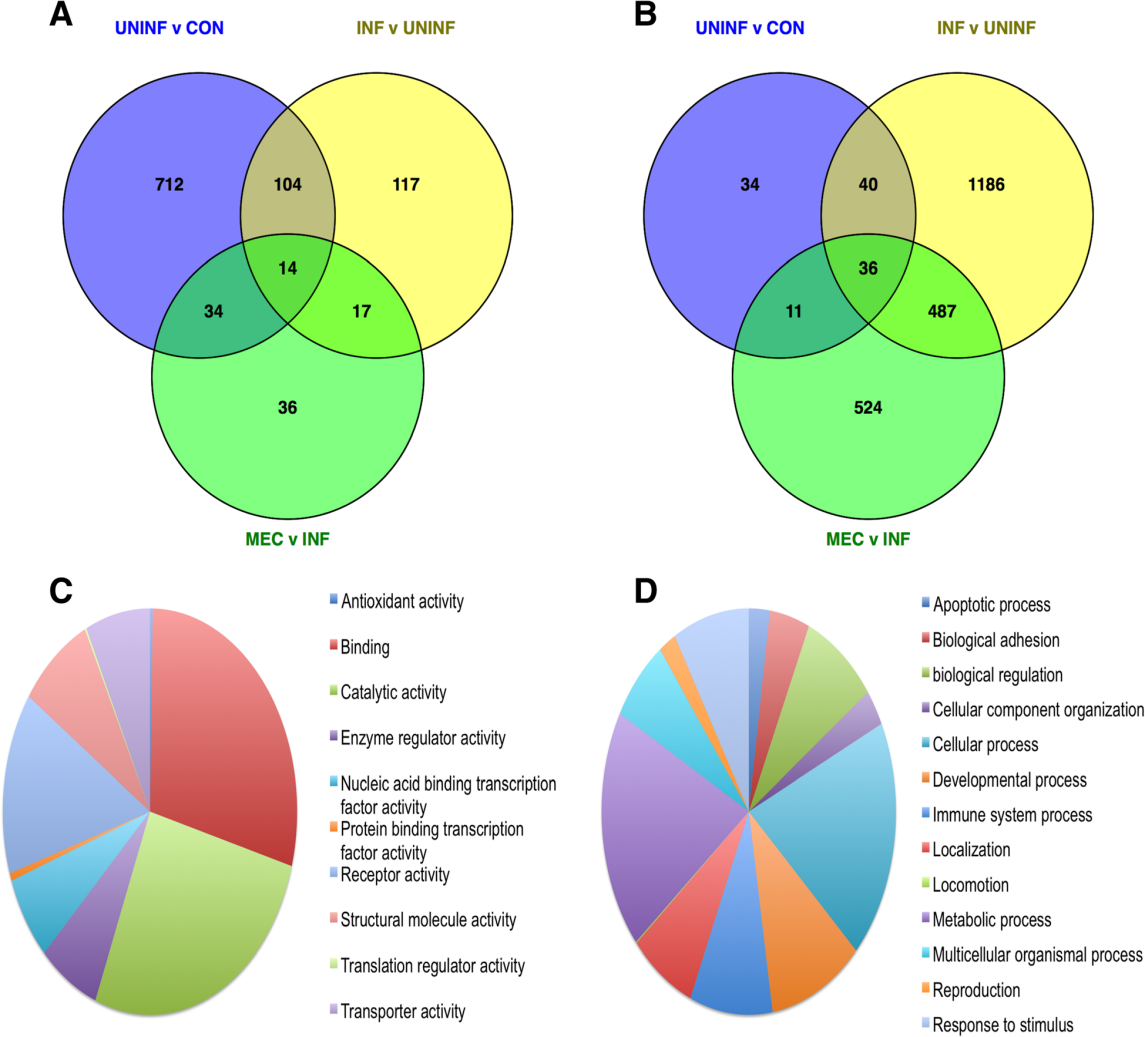
Overview of differential gene expression in PRRSV-inoculated reproductive tract. **a** and **b** Venn diagrams showing the numbers of differentially expressed genes (DEGs) in endometrium (**a**) and fetal thymus (**b**) for each of the three between-group contrasts (UNINF v CON, INF v UNINF, and MEC v INF). **c** and **d** Pie charts of DEGs identified across all six contrasts grouped by molecular activity (**c**) and biological process (**d**) terms

Three contrasts were made for each tissue type: UNINF v CON, INF v UNINF, and MEC v INF. For endometrium, the numbers of DEGs for each contrast were 864, 252, and 101 respectively, and for thymus the total numbers were 121, 1749, and 1058 respectively (Additional file 2). Some genes were common to multiple contrasts and the distribution of genes across contrasts is shown in Figs. 2a and b. In total, 1034 DEGs were identified in endometrium and 2318 in thymus, with 428 genes differentially expressed in both tissues. Combined endometrium and thymus DEGs were classified by GO molecular function and biological process terms using PANTHER (Figs. 2c and d). The five most common molecular process terms were: ‘binding’ (29.0 %), ‘catalytic activity’ (26.7 %), ‘receptor activity’ (14.5 %), structural molecule activity (8.1 %), and ‘transporter activity’ (7.1 %). The top five biological process terms were: ‘cellular process’ (19.1 %), ‘metabolic process’ (19.1 %), ‘developmental process’ (10.4 %), ‘immune system process’ (9.1 %), and ‘response to stimulus’ (8.4 %). A more detailed investigation of the individual contrasts was also carried out using IPA and WGCNA. These results are presented in the following sections, and all the data included in Additional files 3, 4, 5, 6. Additional file 3 contains the IPA results from the edgeR analyses; Additional file 4 contains the module-trait correlations and significance for each WGCNA contrast; Additional file 5 contains the gene significance and module membership values for each WGCNA contrast; Additional file 6 contains the IPA results from the WGCNA analyses.

### RT-qPCR validation of RNA-seq data

RNA-seq expression data for specific genes of interest was validated by RT-qPCR. Endometrial expression was tested for 12 genes and thymic expression for 11 genes. There were 16 genes in total (7 genes were tested on both tissues). Genes were selected on the basis of differential expression in at least one contrast from the RNA-seq analyses or membership of a significant ‘WGCNA’ module. The genes chosen were annotated as being associated with biological functions, pathways, or upstream regulators of interest identified by ‘IPA’ and discussed in detail in subsequent sections. They consisted of one immunoglobulin gene *(IGJ*), three T or NK cell marker genes (*CD3D*, *CD8B*, and *GZMA*), two TWIST1-regulated genes (*FBN2* and *PAMR1*), five interferon-regulated genes (*CXCL10*, *GBP1*, *ISG20*, *MX1*, *OAS1*), four inflammation-associated genes (*CASP1*, *CCL2*, *ITIH4*, and *MPO*), and one apoptosis-associated gene (*TNFSF10*).

A total of 19 cases of differential expression in endometrium were found by RNA-seq for the 12 genes across each of the 36 tests (12 genes x 3 contrasts); for thymus, 14 cases of differential expression were found for the 11 genes across 33 tests (11 genes x 3 contrasts). Confirmation of differential expression by RT-qPCR required a significant difference (*P* < 0.05) in gene expression between the two groups in the contrast tested. A comparison of the expression differences and their statistical significance identified for individual tests by RNA-seq and RT-qPCR methods is shown in Additional file 7. In endometrium, differential expression was confirmed for 10 out of 19 cases identified by RNA-seq, however the expected direction of expression (i.e. which group in the contrast exhibited the greater expression of that gene) was confirmed for all 19 cases. In thymus, differential expression was confirmed for all 14 cases.

There were also four cases (two in each of endometrium and thymus) of genes that belong to significant WGCNA modules, but that weren’t identified individually as being differentially expressed by the ‘edgeR’ analysis. RT-qPCR confirmed the direction of expression for each of these genes.

**Fig. 3:**
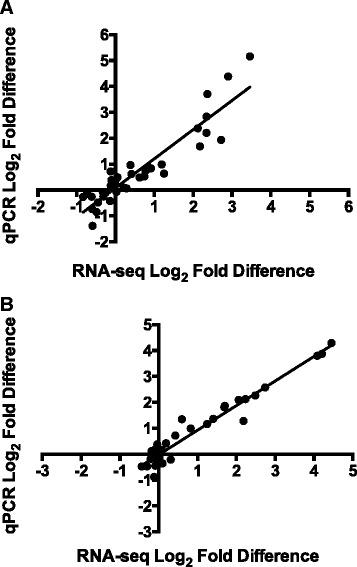
Concordance of expression data obtained by RNA-sequencing and Reverse transcription quantitative polymerase chain reaction (RT-qPCR). Scatter plots of log_2_-transformed fold differences obtained from RNA-sequencing (x axis) or RT-qPCR (y axis) methods for selected genes tested by RT-qPCR in endometrium (**a**) or thymus (**b**). Overall, there was good agreement in expression values determined by each method. Linear regression lines are shown for each tissue. The slope values are close to 1 for both endometrium and thymus (1.125 and 0.949 respectively)

Overall, there was good concordance between expression values calculated by RNA-seq and RT-qPCR, as shown in Fig. 3. This strengthens confidence in the reliability of these expression differences and their interpretation, and is in agreement with previous studies that have documented the similarity in expression data produced by the two methods [30, 31].

### Comparison of UNINF v CON endometrium transcriptome

**Fig. 4:**
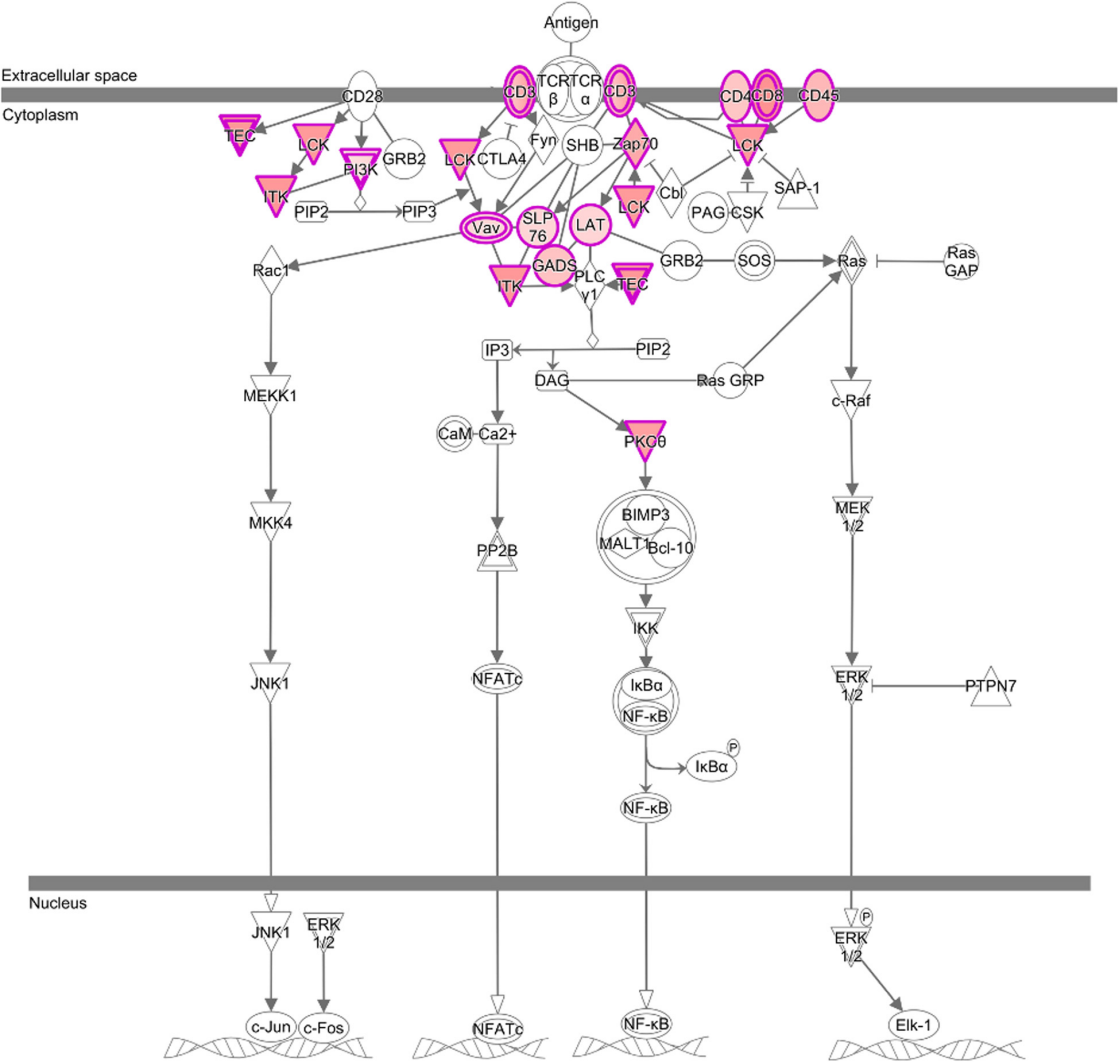
T Cell Receptor (TCR) signaling pathway activation in endometrium of UNINF fetuses. Image of the canonical pathway of T cell receptor signaling from Ingenuity Pathway Analysis (IPA) highlighting genes found to be differentially expressed in UNINF v CON endometrium. The red color indicates that the gene was upregulated in UNINF endometrium whereas green color signifies downregulation

The expression profile of the endometrium of UNINF in comparison to CON fetuses was predominantly characterized by an adaptive immune response to PRRSV infection. Fifteen of the top 25 most upregulated genes in the UNINF v CON contrast were immunoglobulin genes, and ‘B cell development’ was one of the most significant canonical pathways enriched for DEGs from this contrast. The importance of T cells at this site of infection was also evident. Specifically, CD28 and iCOS-iCOSL signaling pathways in T helper cells were the two most significant canonical pathways, and both were activated in UNINF endometrium. The TCR signaling pathway and DEGs that map to it are shown in Fig. 4. DEGs that belong to these pathways are class II antigen presentation genes *SLA-DMA*, *SLA-DQA1*, *SLA-DQB1*, *SLA-DRA1*, and *SLA-DRB1*, and T cell signaling genes *CD3D*, *CD3E*, *CD3G*, *CD4*, *ICOS*, *GRAP2*, *LCK*, and *ZAP70*. Cell surface marker genes of cytotoxic T lymphocytes (CTL) and NK cells were also highly upregulated in UNINF endometrium, including the cell surface receptors *CD8A*, *CD8B*, and *SLAMF7*, and the cytolytic enzymes *GZMA*, *GZMB*, *GZMK*, and *GNLY*. Biological functions that were increased or activated in UNINF endometrium include ‘Quantity of T lymphocytes’, ‘T cell migration’, and ‘Activation of T lymphocytes’.

The adaptive nature of the immune response to infection in UNINF endometrium reflected the 21-day duration of the infection period in the gilt. Most pigs infected with PRRSV produce antibodies within 14 days post-inoculation, and so the expression of immunoglobulin genes in this context was not unexpected [32]. These early antibodies, however, are not protective against PRRSV and the appearance of neutralizing antibodies is delayed until at least 4 weeks post-infection [33]. Indeed, these early antibodies have been reported to enhance infection by facilitating entry of the virus into target macrophage cells. This antibody-dependent enhancement of infection (ADEI) is mediated by the FcγRIIb receptor [34], whose gene is upregulated in UNINF endometrium samples. The UNINF endometrial expression profile also indicated an ongoing T helper cell response to infection, coupled with a cytolytic response that could be driven either by CTL, NK cells, or both. The first appearance of PRRSV-specific IFN-γ secreting T cells in infected pigs is typically around 2–3 weeks, and so it is feasible that PRRSV-specific CTL could contribute to PRRSV immunity at this site [32, 35]. Specific cell-mediated immune responses against PRRSV are generally considered to be weak, but much of the evidence for this comes from peripheral blood rather than the infected tissues where effector cells would be preferentially targeted in comparison to circulating naive lymphocytes [35, 36]. NK cells are known to increase in numbers in the endometrium of PRRSV-infected sows [37], but, as with CTL, PRRSV can suppress their cytotoxicity, at least *in vitro* [38].

Evidence for an innate response to viral infection in UNINF endometrium was also observed, albeit at a lower level than the adaptive response. ‘Granulocyte adhesion and diapedesis’, and ‘Complement system’ were among the top 20 most significant canonical pathways enriched for DEGs. ‘Interferon signaling’, an important pathway in the innate immune response to viral infection, was significantly enriched for DEGs and active in UNINF endometrium, but was not in the top 20 pathways. However, IFN-γ and IFN-α were activated in UNINF endometrium. Also, the functions ‘Cell movement of phagocytes’ and ‘Inflammatory response’ were increased in UNINF endometrium.

WGCNA identified 10 modules whose expression profiles were significantly correlated with the UNINF group. Two of these were of particular biological interest. Gene expression in the brown module had the highest positive correlation to the UNINF group. Forty-four percent of the genes it contains were identified as DEGs by ‘edgeR’, and the most significant biological functions, pathways, and upstream regulators are all associated with lymphocyte-based immune responses. This module therefore provided independent validation of the results of the edgeR analysis for this contrast. Gene expression in the purple module was negatively correlated with the UNINF group. TWIST1, a transcription factor that is predominantly expressed in placental tissue, was the most significant upstream regulator of the purple module genes, and was inhibited in the UNINF compared to CON group. The module was enriched for genes involved in angiogenesis and musculoskeletal development that could adversely affect endometrial and placental function, and subsequent fetal development. Indeed, the biological function attributes ‘Litter size’ and ‘Fertility’ were inhibited in the UNINF group.

### Comparison of INF v UNINF endometrium transcriptome

Two principal differences were found between INF and UNINF transcriptomes in endometrium. The first was an increase in type I interferon signaling and innate antiviral responses; the second was a decrease in adaptive immune responses, particularly of T cell responses, in INF individuals. ‘Interferon signaling’ was the most significant canonical pathway enriched for DEGs for this contrast, and was predicted to be more active in INF endometrium. Among the upregulated genes in this pathway were the signal transduction molecules *IRF9*, *STAT2*, and *SOCS1*, and antiviral effector molecules *MX1* and *OAS1*. ‘Antiviral response’ was the most significant biological function among the DEGs for this contrast, and was activated in INF endometrium. Associated innate antiviral functions such as ‘Phagocytosis by macrophages’ and ‘Immune response of antigen presenting cells’ were also significant and increased in INF endometrium. The most significant upstream regulators were the type I interferon gene *IFNA2* and interferon regulatory factor *IRF7*.

The strong upregulation of type I interferon stimulated genes in the endometrium of INF fetuses may seem odd given the reputation PRRSV has for subverting and suppressing type I interferon responses. However, while it is certainly the case that PRRSV can inhibit IFN-α production by alveolar macrophages *in vitro* [39], *in vivo* production of IFN-α has been reported following infection with several PRRSV isolates, including the blood of gilts from this disease model [40, 41]. It is clear that some cell types, for example plasmacytoid dendritic cells, retain their ability to secrete IFN-α during PRRSV infection [42]. Another counterintuitive aspect of this interferon signaling is that despite its antiviral action, in this instance it was positively associated with transmission of the virus to the fetus. One explanation is that this is simply a reflection of the greater viral load in the INF compared to UNINF endometrial samples. The interferon response pathway is directly activated by a number of cellular pattern recognition receptors for RNA viruses, such as TLR3, TLR7, and RIG-I, and so the expression of interferon-stimulated genes (ISGs) in infected cells is positively associated with the amount of viral RNA in the cell [43]. Also, increased expression of these genes may actually be detrimental, as IFN-α can upregulate sialoadhesin gene expression in macrophage cells [44]. The presence of sialoadhesin on the cell surface is known to be important for PRRSV entry into the cell [37]. Despite its antiviral action, levels of IFN-α in gilts tended to be positively associated with fetal mortality rate in the large challenge model, and could be a good indicator of viral load and disease severity [41].

**Fig. 5:**
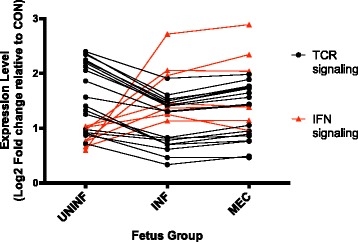
Relationship between interferon and T cell receptor signaling in endometrium during PRRS progression. Scatter plot of mean expression values of individual genes from the interferon and T cell receptor canonical signaling pathways in Ingenuity Pathway Analysis in UNINF, INF, and MEC fetuses relative to expression in CON fetuses

TCR signaling and iCOS-iCOSL signaling in T helper cells were among the top 5 most significant canonical pathways, and all of the DEGs that mapped to those pathways were downregulated in the INF group. The biological function attributes ‘Quantity of CD4+ lymphocytes’ and ‘Quantity of T lymphocytes’ were decreased in the endometrium adjacent to INF fetuses. Similarly, the terms ‘Apoptosis of leukocytes’ and ‘Cell death of T lymphocytes’ were increased in the INF group. The relationship between interferon and TCR signaling across UNINF, INF, and MEC groups is depicted in Fig. 5.

The reduction in lymphocyte signaling in endometrium in association with fetal infection is a significant finding. One possibility is that this was caused by apoptosis. Although lymphocytes are not permissive to PRRSV, bystander apoptosis of uninfected lymphocytes in PRRSV-affected tissues, including endometrium, has previously been documented and was observed in endometrial samples in this infection model [16, 18, 29, 45]. Some of the DEGs in the INF v UNINF contrast have been implicated in lymphocyte apoptosis. *SOCS1*, for example, which is upregulated in INF endometrium, causes an increase in apoptosis when constitutively expressed in the T cell lineage of mice [46]. High expression of the cytokine *CXCL10* in blood is associated with T lymphocyte apoptosis in chronic hepatitis C infected individuals [47]. Some of the downregulated genes in INF endometrium encode cell membrane markers of the T cell lineage such as CD3E and CD8B. All together, the data suggest that a combination of high viral load and reduction in T cell immune responses at the endometrium created favorable conditions for viral transmission to the fetus.

Three gene co-expression modules were significantly correlated with the INF group, and two of these are of particular biological interest. The red module was positively correlated with gene expression in the INF group, and was principally enriched for genes involved in interferon signaling, in agreement with the edgeR analysis. The dark red module was negatively correlated with gene expression in the INF group, and was enriched for genes regulated by TWIST1, as seen with the previous contrast.

TWIST1 is a transcription factor that controls the invasion of the trophoblast layer into maternal tissues during placentation in humans, and whose expression is associated with placental implantation in cattle [48–50]. Dysregulation of this transcription factor’s activity would likely have detrimental consequences for placental attachment, development and normal function. Indeed, histological analysis of 120 endometrium samples from the large-scale experiment identified the separation of endometrial and placental epithelium ranging from mild, multifocal areas to severe, diffuse regions that exhibited marked necrosis of chorionic villi and damage to blood vessels [18]. PRRSV has a restricted tropism for CD163^+^ and Sn^+^ macrophages, and so it is unlikely that it can pass through the multiple cell layers of an intact maternal-fetal interface by spreading between different cell types. However, if this barrier was disrupted, free and macrophage associated virus may be more likely to cross. It is possible that downregulation of TWIST1 at the maternal-fetal interface in UNINF compared to CON and again in INF compared to UNINF fetuses is involved in transmission of the virus from the endometrium to the placenta and ultimately into other fetal tissues. Alternatively, the histological lesions and downregulation of TWIST1 could occur following transmission rather than preceding it, as a consequence of placental infection, in which case they could contribute to placental dysfunction and fetal pathology rather than transmission. This would fit with an alternative proposed mechanism of virus transmission, in which infected macrophages migrate by diapedesis through intact uterine and placental cell layers. In support of this mechanism, others have observed virus-positive cells in close proximity to undamaged maternal-placental interface by microscopy [29].

### Comparison of MEC v INF endometrium transcriptome

The final endometrium contrast, MEC v INF, was conducted to identify changes in the endometrium transcriptome associated with disease progression in the fetus from the VIA to MEC preservation categories. As previously described, meconium staining was almost exclusively observed in the PRRSV inoculated litters and is indicative of impending fetal death [17]. The canonical pathways ‘Granulocyte adhesion and diapedesis’ and ‘Agranulocyte adhesion and diapedesis’ were the top canonical pathways. Upregulated DEGs that map to these pathways include the pro-inflammatory chemokines *CCL2*, *CCL3L1*, *CCL4*, and *CCL8*. These same genes were also annotated to significant biological function terms that included ‘Chemoattraction of monocytes’, ‘Chemotaxis of myeloid cells’ and ‘Chemotaxis of phagocytes’. The most significant upstream regulator identified was the pro-inflammatory transcription factor NF-κB.

No WGCNA modules were significantly correlated with the MEC group for this contrast, and it also had the lowest number of differentially expressed genes of all contrasts. No further dowregulation of TWIST1 was observed between INF and MEC fetuses either. This was an unexpected finding given the prevailing hypothesis that placental lesions play a critical role in fetal pathology and mortality. However, the inflammation-associated nature of the DEGs that were identified is consistent with a host response to PRRSV-induced damage to the placental attachment site. Greater numbers of apoptotic and possibly necrotic cells are observed at the maternal-fetal interface in PRRSV-inoculated compared to mock-inoculated control sows [29]. The fact that PRRSV-associated lesions were found at the placental attachment sites of all 3 groups (UNINF, INF, and MEC) of fetuses from infected gilts in this study may indicate that the majority of expression changes associated with lesion development precede the onset of observable meconium-staining. Also, the potential contribution of PRRSV replication within the fetus to fetal pathology is discussed in a later section.

### Comparison of UNINF v CON thymus transcriptome

**Fig. 6:**
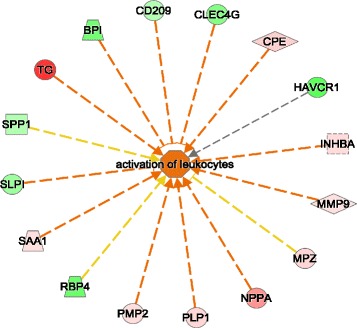
Expression of genes that function in leukocyte activation in uninfected fetuses. Expression of differentially expressed genes from the UNINF v CON contrast in fetus that are annotated with the term ‘activation of leukocytes’ in Ingenuity Pathway Analysis (IPA). Gene color indicates strength and direction of expression change: red color represents upregulation of gene expression in UNINF group; green color represents downregulation of gene expression in UNINF group. Arrow color indicates whether the observed direction of expression for that gene and its potential effect on leukocyte activation is consistent with what would be expected based on experimental evidence curated in the Ingenuity Knowledge Base. An orange line indicates that the direction of expression of that gene is consistent with activation. A yellow line indicates that the direction of expression of that gene is consistent with inhibition. A grey line indicates no known relationship. The predominance of orange arrows reflects the fact that this function exhibited a tendency to be activated by biological function analysis in IPA (Z score of 1.967)

Although no viral RNA was detected in the thymus of any of the UNINF fetuses by RT-qPCR, subtle differences in the thymus transcriptome of UNINF compared to CON fetuses were evident. The biological function annotation term ‘Activation of leukocytes’ was significantly enriched for DEGs and close to being increased in UNINF fetuses (Z score of 1.967). A network of the DEGs associated with this function and their predicted effects based on their expression profile are presented in Fig. 6. Several inhibitors of leukocytes were downregulated in UNINF fetal thymus, such as the lectin genes *CLEC4G* and *CD209*, whereas activating molecules were generally upregulated, including *MMP9*, *NPPA*, and *SAA1*. ‘Calcium signaling’ and ‘Agranulocyte adhesion and diapedesis’ were among the top canonical pathways enriched for DEGs for this contrast. Both pathways contain the myosin genes *MYL4*, *MYL7*, and *MYH11*. No interferon-regulated genes were differentially regulated in UNINF and CON fetuses.

Three WGCNA modules were identified as significantly positively correlated with UNINF fetuses for this contrast, two of which are of particular interest. The green-yellow and purple modules were enriched for pathways and functions that relate to inflammatory responses. The top canonical pathways for the green-yellow module was ‘Hepatic fibrosis’ while the top 3 pathways for the purple module were ‘Granulocyte adhesion and diapedesis, ‘Agranulocyte adhesion and diapedesis’, and ‘Atherosclerosis’. Inflammation-associated cytokine genes were also among the top upstream regulators of DEGs: *TGFB1* and *IL1B* for the green-yellow module, and *IL1B*, *TNF*, and *IL6* for the purple module.

None of the genes identified as being differentially expressed between UNINF and CON fetal thymus belonged to innate antiviral defenses activated in the presence of virus. This suggests that the UNINF fetuses were genuinely uninfected, but the presence of small amounts of virus below the detection threshold of the PCR assay cannot be formally excluded. Nevertheless, expression differences in a small number of immune genes were detected, and appeared to have functional relevance. Several pro-inflammatory signaling molecule genes were upregulated, and inhibitory genes downregulated in UNINF fetuses. Leukocyte activator genes included *NPPA*, a cytokine that can activate and induce the mobilization of polymorphonuclear leukocytes, the acute phase protein *SAA1*, and *INHBA*, a secreted protein that can activate resting macrophages [51–53]. Among the downregulated inhibitors are two genes that modulate the responsiveness of macrophages to microbial lipopolysaccharide, *BPI* and *SLPI*, and two inhibitory genes that encode PRRs on macrophages and dendritic cells, *CLEC4G* and *CD209* [54–57]. Some of these genes (*SAA1*, *CLEC4G*, and *CD209*) were also differentially expressed in the thymus of INF fetuses, but others were not (*SLPI*, *NPPA*, and *INHBA*) or changed expression in the opposite direction (*BPI*). This suggests that these transcriptional changes are an early indication of infection in the endometrium impacting on the fetus rather than a direct consequence of fetal infection. Whether this low-level activation of the innate immune system in UNINF fetuses is detrimental or beneficial to survival is unclear. It could be that these changes represent the first signs of a hypoxic response to placental injury, as inflammation and hypoxia are closely linked processes [58]. On the other hand, variation in the extent to which UNINF fetuses are ‘primed’ for infection *in utero* could influence its response to a subsequent challenge, in combination with other genetic and environmental factors.

### Comparison of INF v UNINF thymus transcriptome

In contrast to the endometrium, the response to infection in fetal thymus was predominantly innate in nature with a smaller accompanying adaptive response. The most significant biological functions predicted to be activated in INF compared to UNINF fetuses included ‘Inflammatory response’, ‘Cell movement of phagocytes’, ‘Activation of phagocytes’, and ‘Antimicrobial response’. The functions and attributes ‘Quantity of T lymphocytes’, ‘Activation of T lymphocytes’ and ‘Priming of T lymphocytes’ were also significant and activated in INF fetuses, but outside the top 100 terms.

The two most significant canonical pathways for the INF v UNINF contrast were ‘Hepatic fibrosis’ and ‘Altered T and B cell signaling in rheumatoid arthritis’, both of which represent inflammatory disease states. Upregulated genes that map to one or both of these pathways include pro-inflammatory chemokines and their receptors (*CCL2*, *CXCL9*, and *CCR5*) and pro-inflammatory cytokines (*TNF*, *IL1B*, *IL1A*). The anti-inflammatory cytokine gene *IL10* and its receptor *IL10RA* were also upregulated in INF thymus, and map to these pathways. Other significant innate inflammatory pathways with upregulated genes included ‘Complement System’, particularly genes encoding components of the classical pathway (*C1R*, *C4A*, *C2*), and ‘Acute Phase Response Signaling’ (*SAA1*, *SAA4*, *HP*, *RBP4*). The pathway ‘Role of pattern recognition receptors (PRRs) in recognition of bacteria and viruses’ was significant due to a variety of upregulated PRRs that included *TLR2*, *TLR3*, *TLR5*, *TLRs7-9*, and *NOD1*. The ‘Interferon signaling pathway’ contained several of the most upregulated genes in this contrast, including *IFIT1*, *MX1*, and *OAS1*. The ‘Antigen Presentation Pathway’ was also significant, with genes involved in both class I (*TAP1*, *TAP2*, *SLA-5*, *SLA-6*, *B2M*) and class II (*SLA-DOB*, *SLA-DRA1*, *SLA-DMA*, *SLA-DMB*, *CIITA*) presentation pathways upregulated in INF thymus. Finally, the ‘Death Receptor Signaling’ pathway was activated in INF thymus. Several pro-apoptotic genes (*FAS*, *TNFSF10*, *CASP1*, *CASP10*, *TIPARP*) belonging to this pathway and were upregulated in INF thymus. The upstream regulator analysis for this contrast identified IFN-α, IFN-γ, TNF- α, and IL1-β as being activated.

Ten WGCNA module expression profiles were significantly correlated with the INF group. The brown module for this contrast was highly positively correlated (*r* = 0.84) with INF fetuses. It was enriched for genes in the ‘Interferon signaling’, ‘Antigen Presentation’, and ‘T helper cell differentiation pathway’. Nodal, regulator genes with high intramodular connectivity that are good candidates for driving this expression included the NK and T cell chemokine *CXCL10* and the transcription factor *STAT1*. The blue module, also positively correlated with INF, contains many of the inflammation-associated DEGs. Nodal genes in this network include the cytokine and growth factor receptors *ACVRL1*, *FGFR2*, and *TNFRSF21*. Two of the most significant modules were negatively correlated with the INF group. The turquoise module was enriched for genes in the ‘Cell cycle’ and ‘DNA damage checkpoint’ pathways, and the salmon module contained genes that function in the translation and processing of RNA. Inhibition of cell proliferation and inhibition of mRNA translation are downstream effects of interferon signaling.

Many of the DEGs in INF thymus map to canonical pathways or are annotated to biological processes carried out by macrophages and dendritic cells. Both cell types are naturally present in the thymus, where they may participate in antigen presentation to thymocytes or engulfment of apoptotic, deleted thymocytes [59, 60], and both cell types are permissive to PRRSV infection, which may explain why the thymus is a primary site of viral replication in the fetus. Several of the most upregulated chemokine genes act on monocytes/macrophages or are produced by activated macrophages, including *CCL2*, *CXCL9*, and *CXCL10*, which probably indicates that additional macrophages migrated to the infected thymus following infection. INF fetuses appear to mount a significant innate immune response against the virus in thymus. Genes downstream of type-I interferon signaling are heavily upregulated, and pro-inflammatory signaling through PRRs and cytokines, the complement pathway, acute phase response signaling, and antigen presentation are all activated in response to PRRSV infection. A number of these genes map to regions of the genome that are associated with fetal resistance to PRRSV, and could help to identify causative single nucleotide polymorphisms (SNPs) [61]. This is the first detailed study of the fetal response to PRRSV infection at the transcriptomic level, although a previous study did find an upregulation of the pro-inflammatory cytokines genes for IFN-γ and TNF-α, which is in agreement with our analyses [16].

Another immunosuppressive strategy of the virus is to promote the host expression of the cytokine IL-10 [62]. Both *IL10* and its receptor *IL10RA* were upregulated in INF thymus, but the majority of upregulated genes were pro-inflammatory in nature, which would be predicted to tip the overall signaling balance towards immune activation rather than suppression.

The predominance of innate antiviral and inflammatory response genes amongst the thymus DEG set contrasts with the mainly adaptive nature of the immune response observed in the endometrium. Given that innate and inflammatory responses precede adaptive responses, the most likely explanation is that the duration of infection in fetuses is shorter than in the gilt. Previous studies that adopted similar inoculation and necropsy time-points in relation to gestational day found that only a small percentage (<5 %) of live fetuses produced detectable antibodies to PRRSV [16, 29]. However, Rowland did observe germinal centers in the lymph nodes of infected fetuses, indicating that the start of an adaptive response to infection was underway in those fetuses [16]. In the present study, a general upregulation of antigen presentation genes was observed in the expression profile of INF thymus.

### Comparison of MEC v INF thymus transcriptome

**Fig. 7:**
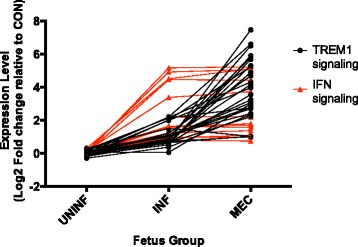
Relationship between interferon and TREM1 signaling in fetal thymus during PRRS progression. Scatter plot of mean expression values of individual genes from the interferon and TREM1 signaling pathways in Ingenuity Pathway Analysis in UNINF, INF, and MEC fetuses relative to expression in CON fetuses

The expression profile of MEC fetuses featured a further upregulation of pro-inflammatory gene expression compared to the INF group. Inflammation was the most significant biological function term, and was increased in MEC fetuses. Just under half (49.4 %) of DEGs in this contrast were also differentially expressed in INF v UNINF fetuses, with the majority of them being upregulated in INF v UNINF, and then further upregulated in MEC v INF. The top six canonical pathways all related to inflammation/inflammatory disorders: ‘Hepatic fibrosis’, ‘Granulocyte adhesion and diapedesis’, Agranulocyte adhesion and diapedesis’, ‘Role of macrophages, fibroblasts, and endothelial cells in rheumatoid arthritis’, ‘Atherosclerosis signaling’, and ‘TREM1 signaling’. Cytokines *IL1A*, *IL1B*, *CCL2*, *CCL4*, *CCL8* and *CXCL8* (*IL8*) are examples of the pro-inflammatory signaling molecule DEGs that map to these pathways. Many of the top 50 upregulated genes are markers of activated neutrophils, macrophages, and NK cells. Examples include both antimicrobial molecules (*AZU1*, *CAMP*, *CHIT1*, *CHI3L1*, *GZMA*, *GZMB*, *GNLY*, *LTF*, and *MPO*) and pro-inflammatory signaling molecules (*CCR2*, *CD177*, *RETN*, *S100A8*, *S100A9*, and *S100A12*). Interestingly, this increase in inflammatory signaling is not associated with a further increase in interferon signaling, which remains at a similar level to that observed in the INF group (Fig. 7).

WGCNA identified four modules whose expression correlated with the MEC group, of which two are of particular biological interest. The inflammation-associated genes mainly fell into the positively correlated turquoise module. The green module, one of two negatively correlated modules, was enriched for genes that map to the ‘T cell signaling’ canonical pathway and are annotated with the biological functional attribute term ‘Quantity of T lymphocytes’. They include T cell surface receptor genes *CD3D*, *CD3E*, *CD3G*, *CD4*, *CD8A*, and *CD8B*. The T cell growth factor *IL2* was not in this module, but was found to be downregulated in MEC thymus.

The transcriptional profile of MEC fetal thymus points to an excessive inflammatory/innate immune response to infection with the potential to damage host tissues. The possible reduction in T cell numbers observed in MEC fetuses could be one consequence. A previous study found that piglets born following congenital infection of PRRSV exhibited a significant depletion of cortical thymocytes in the thymus, at least in part due to higher rates of apoptosis [63]. Overall, over 1000 genes were differentially expressed in this contrast, and the majority of the most highly upregulated genes are associated with the signaling and effector functions of phagocytic cells. Many of these genes, such as pro-inflammatory signaling molecules *RETN*, *S100A8*, *S100A9*, and *S100A12*, have previously been identified by transcriptomic studies as being highly upregulated in pigs exhibiting infectious disease pathology [10, 64, 65]. This includes pigs infected by the highly pathogenic PRRSV (HP-PRRSV) variant, a strain of the virus originally identified in China that induces an excessive release of pro-inflammatory cytokines and widespread tissue damage and cell death. These dramatic changes at the transcriptional level, however, are not associated with the development of any PRRSV-associated microscopic lesions in MEC thymus tissue itself. The relative lack of obvious macroscopic or microscopic lesions in the thymus and other fetal tissues examined, and their relative abundance at placental attachment sites, has led most researchers to conclude that events at the maternal-fetal interface likely drive disease progression in the fetus [16, 66]. A similar distribution of lesions was also observed in our large-scale challenge model [18], and none of the fetuses in the RNA-seq subset chosen for this study had observable lesions in fetal thymus. At the molecular level though, we observed striking changes in gene expression of MEC compared to INF fetal thymus, but only minor changes in endometrial gene expression.

The question of whether this thymic expression profile is primarily a consequence of virus replication in the fetus or a hypoxic response to placental injury is difficult to resolve on the basis of gene expression data alone. Inflammation and hypoxia are closely related processes that are frequently both present in pathologic lesions. Furthermore, hypoxia can either be the cause of inflammation, for example following ischemia, or a consequence of it, such as at sites of infection or in atherosclerotic plaques [58]. Hypoxia can also lead to the suppression of TCR signaling in the thymus [67]. The cellular response to hypoxia is controlled by the transcription factor HIF-1α [68]. Some features of HIF-1α activation were observed in the thymic expression profile of MEC fetuses, such as the upregulation of genes that promote angiogenesis (e.g. *ADM*, *PDGFA*, *PDGFB*) and erythropoiesis (e.g. *HBB*, *HMOX1*). However, there was no evidence of an upregulation of enzymes and transporters in the glyocolytic pathway, a key metabolic response to low oxygen availability that is activated by HIF-1α. Given the small differences in endometrial compared to thymus gene expression between INF and MEC fetuses and the large amounts of viral RNA found in almost all MEC fetuses, it is likely that infection within the fetus does makes a significant contribution to disease progression and ultimately, fetal death.

## Conclusions

Contrary to our original hypothesis, the immune responses to PRRSV infection at the maternal/fetal interface and in the fetus were found to be substantially different. In the endometrium, the expression profile indicated a predominantly adaptive immune response to infection with both antibody and cell-mediated immune components. On the other hand, an innate, interferon-led response accompanied by an inflammatory response regulated by cytokines that include IFN-γ, IL-1β and TNF-α predominated in infected fetal thymus. A difference in the duration of infection at each site is the most likely explanation for this. Two potentially significant characteristics of the endometrial expression profile of infected versus uninfected fetuses were determined, which could relate to virus transmission. The first was an increase in viral load and decreased T cell signaling; the second was a downregulation of genes controlled by the transcription factor TWIST1, known to be important for placental development and implantation. Whether TWIST1 dowregulation and lesion development precede or follows virus transmission to the placenta though cannot be determined. Finally, and again contrary to our original hypothesis, we found that fetal pathology is most likely influenced by events occurring at both the fetal attachment site and within the PRRSV-infected fetus. Thymus samples of fetuses exhibiting gross external signs of pathology (meconium-staining) exhibited an increase in the expression of cytokine and granulocyte genes previously implicated in severe inflammation associated with swine infectious disease pathology.

## Availability of supporting data

The RNA-seq data supporting the results of this article are available in the Geo database (www.ncbi.nlm.nih.gov/geo) under series identifier GSE71205.

## Additional files

Additional file 1: List of primer and probe sequences. Word (.docx) file containing a table of primer and probe sequence information for RT-qPCR assays. (DOCX 20 kb) Additional file 2: Lists of differentially expressed genes. Excel (.xlsx) file of the differentially expressed genes (DEGs) identified by the edgeR software from the six contrasts of the transcriptomic analysis. Results for each contrast are on a separate tab (6 tabs in total). (XLSX 656 kb) Additional file 3: Gene function enrichment analysis results on DEG lists. Excel (.xlsx) file of three function enrichment analyses from the IPA software: biological function (BF), canonical Pathway (CP), and upstream regulator (UR), for each of the six DEG lists (18 tabs in total). (XLSX 801 kb) Additional file 4: WGCNA module trait correlations. Excel file (.xlsx) of the modules identified for each of the six contrasts by the WGCNA software, their correlation with the traits of interest, and the statistical significance of that correlation (6 tabs in total). (XLSX 70 kb) Additional file 5: Gene significance and module membership results. Excel file (.xlsx) of the gene significance and module membership results from the WGCNA analysis for each of the six contrasts (6 tabs in total). (XLSX 1631 kb) Additional file 6: Gene function enrichment analysis results on WGCNA data. Excel (.xlsx) file of three function enrichment analyses from the IPA software: biological function (BF), canonical Pathway (CP), and upstream regulator (UR), for each of the modules from the six contrasts mentioned in the Results section (36 tabs in total). (XLSX 7834 kb) Additional file 7: Comparison of RNA-seq and RT-qPCR expression data for individual genes. Excel (.xlsx) file of log_2_ transformed fold differences, for both RNA-seq and RT-qPCR, for genes used for RT-qPCR validation of RNA-seq data from endometrium and thymus (2 tabs in total). (XLSX 37 kb)
