# Supplementary material for: Genome-wide analysis of the transcriptional response to porcine reproductive and respiratory syndrome virus infection at the maternal/fetal interface and in the fetus
Source: BMC Genomics. 2016 May 20;17:383. doi: 10.1186/s12864-016-2720-4 (PMC4875603; doi:10.1186/s12864-016-2720-4)
Supplement: Additional file 1: — List of primer and probe sequences. Word (.docx) file containing a table of primer and probe sequence information for RT-qPCR assays. (DOCX 20 kb) [file 12864_2016_2720_MOESM1_ESM.docx]

| **Gene**  **symbol** | **PCR-product**  **(bp)** | **Primer sequence** | **Probe sequence** |
| --- | --- | --- | --- |
| *CASP1* | 98 | 1088F: CCTGTGGCCTGGAGGAAAT  1169R: TCGGCAGTGGGCATCTG | 1114T: ATCTGCCAGCTCAAATGAAAATCGAACCTT |
| *CCL2* | 93 | 190F: GCGGCTGATGAGCTACAGAAG  263R: CCGCGATGGTCTTGAAGATC | 213T : TCACCAGCAGCAAGTGTCCTAAAGAAGCA |
| *CD3D* | 115 | 147F: AGGAACAGAAGGAGAACTACTTTCAGA  239R: TTTGGCGCATTGCACTTATATAA | 186T: CCTGGGAAAACGCATCCTGGATCC |
| *CD8B* | 107 | 408F: GGCTGAGTGTGGTTGATGTCTTT  490R: TGGTAATCGGCATATTTTCTTCTTG | 432T: CCACCACTGCCCAGCCCACC |
| *CXCL10* | 104 | 174F: TTGAAATGATTCCTGCAAGTCAA  254R: GACATCTTTTCTCCCCATTCTTTT | 198T: CTTGCCCACATGTTGAGATCATTGCCAC |
| *FBN2* | 98 | 816F: GGTATCACTGTGGATGGAAGAG  893R: CAGCGGTAACTACCACGTAAA | 841T: TCAATGAGTGTGCTTTGGATCCTGACA |
| *GBP1* | 141 | 57F: TCAAACCCTCCCGTGAAACA  179R: CACTGTGGTTCGGGCATGT | 141 rev T: CCCGGGTCTCATTGAGAGTTCTTTCCTCT |
| *GZMA* | 144 | 279F: GGAGCTCACTCGATAACCAAGAAA  396R: GCTTTAGAAGTTTAAGGTCACCCTCAT | 341T: TCCTTATCCATGCTTTGACCAGGACACAC |
| *IGJ* | 106 | 217F: TGTAAGTGTGCCCGGATTACTTC  297R: TCTGATGTTTCTCTCCACAATGTCTT | 241T: AGGATCATCCGCTCTGCTGAAGACCC |
| *ISG20* | 169 | 159F: GGGCGAGATCACTGACTACAG  306R: GAAGTCGTGCTTCAGGTCATGA | 273 rev T: CTTGCCTTTCAGGAGCTGCAGGATCTC |
| *ITIH4* | 89 | 675F: CCCAGAGCAGCAGGAAACA  741R: GACTGTCCGGTTCACATCATAGC | 696T: CCTGGATGGCAACTTCATCGTCCG |
| *MPO* | 125 | 18F: TGGAGGGTAGTGCTGGAAGGT  118R: ATCTCATCTACCACGATTTGGTTCT | 52T: TCCTCCGGGGCCTCATGGC |
| *MX1* | 107 | 1838F: CAGCACCTGATTGCCTACCA  1924R: GGTCCGGAGGATGAAGAACTG | 1871T: AAGCGCATCTCCAGCCACATCCCT |
| *OAS1* | 110 | 330F: GCGCCGAGGAGAATTCATC  416R: TGGACCTCAAACGTCACTTTAAAC | 385 rev T: CTCTTTGACAGGCTTCCAGCTGTCTCC |
| *PAMR1* | 87 | 220F: GTGTCGGGAGTGTTGTGAATA  286R: CTGCAGCAAGGGATGGTATAA | 259T: CTGCCCTGGAAAGAAGGAAGTGGT |
| *TNFSF10* | 130 | 277F: TGTGAGAAAGATGATTTTGAGAACCT  380R: CTCTCTGTGGACCTTTTTCTCTTTCTA | 326T: TCAGAAAAGCAACAAGGCATTCCTCACCT |
| *RPL32* | 118 | 90F: TGGAAGAGACGTTGTGAGCAA  183R: CGGAAGTTTCTGGTACACAATGTAA | 126T: ATTTGTTGCACATTAGCAGCACTTCAAGCTC |
